# Electrophysiology of human cardiac atrial and ventricular telocytes

**DOI:** 10.1111/jcmm.12240

**Published:** 2014-01-28

**Authors:** Jingwei Sheng, Winston Shim, Jun Lu, Sze Yun Lim, Boon Hean Ong, Tien Siang Lim, Reginald Liew, Yeow Leng Chua, Philip Wong

**Affiliations:** a Research and Development Unit, National Heart Centre SingaporeSingapore; b Duke-NUS, Graduate Medical SchoolSingapore; c Department of Cardiothoracic Surgery, National Heart Centre SingaporeSingapore; d Department of Cardiology, National Heart Centre SingaporeSingapore

**Keywords:** Telocyte, potassium channel, hydrogen sulphide, fibrosis

## Abstract

Telocytes (TCs) with exceptionally long cellular processes of telopodes have been described in human epicardium to act as structural supporting cells in the heart. We examined myocardial chamber-specific TCs identified in atrial and ventricular fibroblast culture using immunocytochemistry and studied their electrophysiological property by whole-cell patch clamp. Atrial and ventricular TCs with extended telopodes and alternating podoms and podomers that expressed CD34, c-Kit and PDGFR-β were identified. These cells expressed large conductance Ca^2+^-activated K^+^ current (BK_Ca_) and inwardly rectifying K^+^ current (IK_ir_), but not transient outward K^+^ current (I_to_) and ATP-sensitive potassium current (K_ATP_). The active channels were functionally competent with demonstrated modulatory response to H_2_S and transforming growth factor (TGF)-β1 whereby H_2_S significantly inhibited the stimulatory effect of TGF-β1 on current density of both BK_Ca_ and IK_ir_. Furthermore, H_2_S attenuated TGF-β1-stimulated KCa1.1/Kv1.1 (encode BK_Ca_) and Kir2.1 (encode IK_ir_) expression in TCs. Our results show that functionally competent K^+^ channels are present in human atrial and ventricular TCs and their modulation may have significant implications in myocardial physiopathology.

## Introduction

Telocytes (TC) are newly identified cells that are found in the interstitial space of many organs, including heart 1, bladder 2, lungs 3 and skeletal muscle 4 and intimately contacting the parenchymal tissues. They have previously been described as interstitial Cajal-like cells in atrial and ventricular myocardium 5,6 and very little was known of their function. Recently, TCs have been increasingly recognized to play key supporting physiological roles in the heart because of the seminal work of Popescu and colleagues 7–9. Telocytes have exceptionally long (10–1000 μm), moniliform cellular processes named telopodes (Tps) with intervening podoms and podomers that form an interstitial network connecting different segments of the heart 10 including those of the epicardium 11,12, myocardium 8,13 and endocardium 14. Their close physical association with cardiac progenitors and cardiomyocytes in the stem cell niche has been suggested to be important in the repair and regeneration of infarcted myocardium 11.

Recently, human myometrial TCs have been reported to express calcium-dependent hyperpolarization-activated chloride inward current 15. However, very limited is known about the electrophysiological activity of cardiac TCs although they have been suggested to act as intercellular signalling intermediary in the myocardium with potential role in cardiac rhythm 11 and atrial fibrillation 16. We have recently showed that hydrogen sulphide (H_2_S) modulated the activity of ion channels in human atrial fibroblasts and suppressed their transformation into myofibroblasts in response to transforming growth factor (TGF)-β1. This may have important implications in TGF-β1-mediated cardiac fibrosis and its associated atrial fibrillation 17. In this study, we present first evidence of electrophysiological properties of human TCs derived from atrial and ventricular myocardium and demonstrate the modulatory role of H_2_S on the ion channel activity.

## Materials and methods

### Telocyte identification

Informed consent was gathered from male patients (*n* = 10) with mean age of 63 ± 8.7 years old who were scheduled for coronary bypass surgery and were hyperlipidemic, managed with anti-hypertensive and diabetic medications. The protocol was approved by institutional review board of Singapore General Hospital that conformed to the Declaration of Helsinki. Atrial appendages were collected as surgical by-product. Human atrial interstitial cells were isolated by mincing the appendages to less than 1 mm^3^ and followed by 0.1% trypsin digestion for 20 min. before plating onto tissue culture-treated 60-mm dishes to produce fibroblastic outgrowth from minced tissue pieces. Outgrown fibroblasts were harvested and re-plated onto a fresh tissue culture dish as passage 1 to isolate homogeneous fibroblast culture that expressed collagen I and fibroblast marker as described previously by our group 18. Atrial fibroblasts were passaged as monolayer in 10% foetal bovine serum supplemented DMEM and antibiotics (100 U/ml penicillin G and 100 μg/ml streptomycin) at 37°C in a humidified atmosphere of 95% air and 5% CO_2_. Human cardiac ventricular fibroblasts (cat no. CC-2904) were acquired from Lonza Inc (Singapore). These cells were isolated from normal adult heart and certified to express greater than 90% collagen I and negative for von Willebrand factor VIII-related antigen. After 3 passages of the atrial and ventricular culture, TCs were distinguishable from other interstitial cells and were typically identified by their characteristic ultra-long moniliform cellular processes of Tps under light microscopy and the cultures from passages 3 to 5 were used for subsequent experiments. These TCs containing cultures were seeded on glass coverslips for immunostaining or in 35 mm dish for whole-cell patch-clamp recordings.

### Immunocytochemistry/Immunohistochemistry

Telocytes were cultured on coverslips, were fixed with 2% paraformaldehyde/PBS for 15 min., permeabilized by 20 min. incubation in PBS containing 0.1% Triton X-100, and blocked in PBS containing 2.5% bovine serum albumin (BSA). Samples were then incubated overnight at 4°C with primary antibodies in PBS containing 0.1% BSA, after which they were incubated for 1 hr at room temperature with secondary antibody. Coverslips were rinsed and mounted on glass slides and examined by using confocal microscopy. Primary antibodies against CD34 (1:10; BD Biosciences, San Jose, CA, USA), c-Kit (1:200; Novus Biologicals, Littleton, CO, USA) and PDGF receptor (PDGFR)-β (1:1000; Cell Signaling, Danvers, MA, USA) were used to identify TCs. Primary antibodies used were anti-Kv1.1 (1/1000; Abcam, Cambridge, MA, USA) to label BK_Ca_ channels, anti-Kv4.3 (1/500; Abcam) to label I_to_ channels and anti-Kir2.1 (1/1000; Abcam) to label IK_ir_ channels. Secondary antibodies used were Alexa Fluor555 (1/1000) and Alexa Fluor488 (1/500)-labelled antibodies (Life Technologies, Carlsbad, CA, USA). Counter staining was performed using DAPI (4′, 6-diamidino-2-phenylindole) to visualize nuclei.

### Electrophysiological recordings

Cell were placed on the stage of a Nikon Diaphot inverted microscope and superfused continuously at 36 ± 1°C with Tyrode solution containing (in mM) 140 NaCl, 5.4 KCl, 1.8 CaCl_2_, 1 MgCl_2_, 10 HEPES and 10 Glucose (pH adjusted to 7.4 with NaOH). The patch-clamped cell was superfused by means of a temperature-controlled micro-superfusor (TC-324B; Warner Instruments, Hamden, CT, USA). Patch pipettes were made from borosilicate glass shanks (Sutter Instrument, Novato, CA, USA) and pulled with a Brown–Flaming puller (Model P-97; Sutter Instrument Co), and had tip resistances of 2–3 MΩ when filled with pipette solution. Pipette tips were polished (Microforge MF830; Narishige, Tokyo, Japan). The patch pipettes were filled with a standard solution containing (in mM) 140 KCl, 1.2 MgCl_2_, 0.05 EGTA, 10 HEPES, 0.1 GTP and 5.0 Mg ATP (pH adjusted to 7.2 with KOH). After a gigaohm seal was obtained by negative pressure suction, the cell membrane was ruptured by a gentle suction to establish whole-cell configuration with a seal resistance >800 MΩ. The cell membrane capacitance (40.3 ± 8.2 pF) was electrically compensated with the pulse software. The series resistance (Rs, 3–5 MΩ) was compensated by 50–70% to minimize voltage errors. Currents were elicited with voltage protocols as described in the following results section for different individual current recordings. Whole-cell voltage-clamp experiments were performed with an Axopatch 200B amplifier (Axon Instruments, Foster City, CA, USA) interfaced to a Digidata 1322A data acquisition system controlled by Clampex version 8.1 software (Axon Instruments). Data were analysed with pCLAMP software (Version 10.0; Axon Instrument) and Origin 8.0 (OriginLab, Northampton, MA, USA).

### Statistical analysis

All data were presented as mean ± SE. Statistical significance of the difference between groups was determined using Student's *t*-test as appropriate. A value of *P* < 0.05 was considered statistically significant.

## Results

### Identification of cardiac telocytes

**Fig 1:**
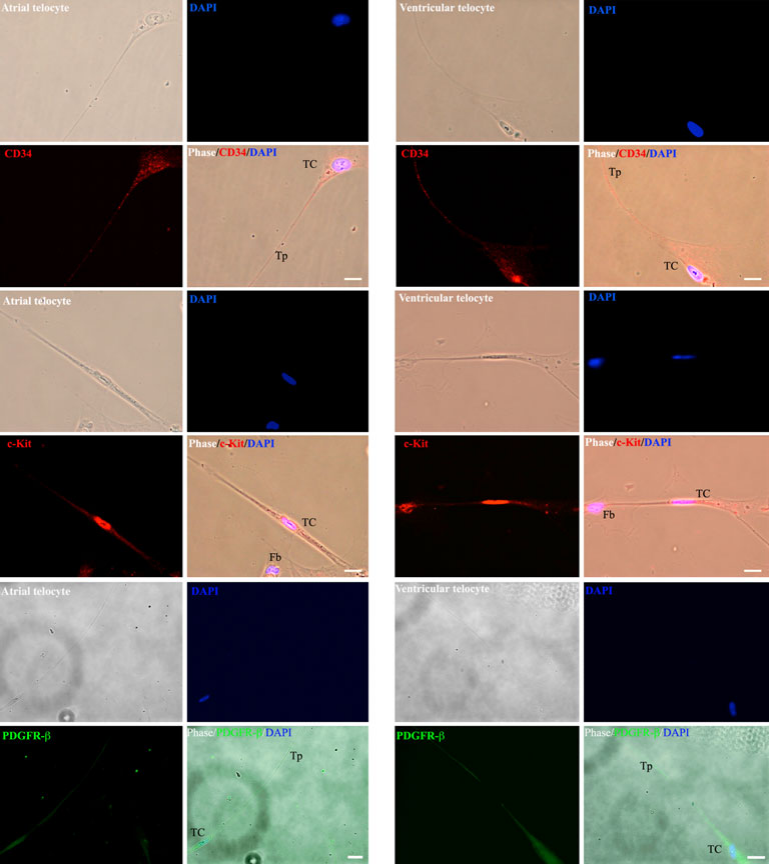
Identification of cardiac atrial and ventricular telocytes (TCs). Immunofluorescent staining for anti-CD34 (red), c-kit (red) and vimentin (green) demonstrated cells with very small cell bodies (∼1:1 ratio cytoplasm to nucleus) and extremely long and thin cellular processes of telopode (Tp) scattered among atrial and ventricular fibroblasts (Fb). TC: telocyte; Fb: cardiac fibroblast; Tp: telopode. Scale bar corresponds to 10 μm.

Telocytes were clearly identifiable in our human atrial and ventricular cardiac fibroblast culture by phase contrast after passage 3. Telocytes displayed characteristic long Tps (100–500 μm) 9 with occasional podomers and podoms that were often in contact with fibroblasts and other TCs 19. In accordance with previous reports 1,8,20, these human TCs stained positive for CD34, c-Kit (CD34/c-Kit double staining in Fig. S1) and PDGFR-β markers (Fig. 1).

### Ion currents in human atrial and ventricular telocytes

**Fig 2:**
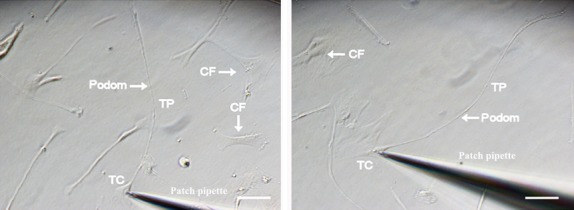
Photomicrographs showing atrial (left) and ventricular (right) telocytes before patch clamping was attempted. TC: telocyte; Fb/CF: cardiac fibroblast; Tp: telopode. Scale bar corresponds to 50 μm.

**Fig 3:**
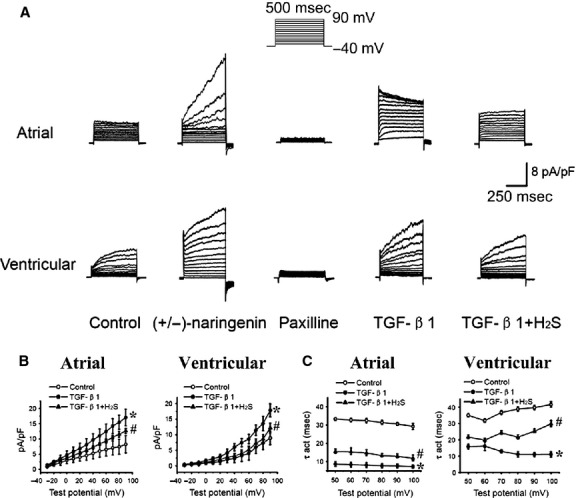
BK_Ca_ currents in human atrial and ventricular telocytes. (A) voltage-dependent current was changed by BK_Ca_ specific blocker Paxilline (100 μM) and opener Naringenin (10 μM) respectively. Effect of H_2_S (100 μM) on BK_Ca_ currents in the presence of transforming growth factor (TGF)-β1 (1 ng/ml). (B) Mean I–V relationship for peak BK_Ca_ in the absence and presence of H_2_S (100 μM) and TGF-β1 (1 ng/ml; *P < 0.05 *versus* basal levels. #*P* < 0.05 *versus* TGF-β1 alone; *n* = 6). (C) Plot of the activation τ (τ_act_) as a function of membrane potential in the presence of TGF-β1 (1 ng/ml) before H_2_S (100 μM) addition (*P < 0.05 *versus* basal levels. #*P* < 0.05 *versus* TGF-β1 alone; *n* = 6).

To record and characterize ion currents in single TCs, cells with long Tps were selected under light microscopy with patch pipettes (Fig. 2) and conventional whole-cell voltage-clamp experiment was performed at 36 ± 0.5°C. Outward currents were activated at depolarization voltages between −30 mV and +90 mV in 10 mV increments from a holding potential of −40 mV. Compared to baseline current density (8.3 ± 2.8 pA/pF, *n* = 6 atrial TCs; 8.9 ± 2.1 pA/pF, *n* = 6 ventricular TCs), the activated currents were sensitive to inhibition (1.8 ± 0.4 pA/pF, *P* < 0.01, *n* = 6 atrial TCs; 2.3 ± 0.5 pA/pF, *P* < 0.01, *n* = 6 ventricular TCs) at +90 mV by 1 mM Paxilline (a specific BK_Ca_ inhibitor) and were responsive (32.4 ± 3.1 pA/pF, *P* < 0.01, *n* = 6 atrial TCs; 20.9 ± 4.2 pA/pF, *P* < 0.01, *n* = 6 ventricular TCs) at +90 mV to stimulation by 10 μM (+/−) Naringenin (a specific BK_Ca_ opener), confirming the presence of BK_Ca_ currents in atrial and ventricular TCs (Fig. 3A). Furthermore, under identical voltage-clamp condition, 1 ng/ml TGF-β1 significantly increased the peak BK_Ca_ current density (17.1 ± 2.7 pA/pF, *P* < 0.01, *n* = 6 atrial TCs; 17.8 ± 2.1 pA/pF, *P* < 0.05, *n* = 6 ventricular TCs) at +90 mV. However, additional presence of H_2_S (100 μM) reduced the effect of TGF-β1 (12.6 ± 0.8 pA/pF, *P* < 0.05, *n* = 6 atrial TCs; 12.2 ± 1.6 pA/pF, *P* < 0.05, *n* = 6 ventricular TCs), thereby demonstrating functionality of the channels and confirming the inhibitory effect of H_2_S on BK_Ca_ currents in TCs (Fig. 3B). Moreover, the rising phase of BK_Ca_ currents at +100 mV with activation τ (τ_act_) at baseline (29.3 ± 1.7 ms, atrial TCs; 41.6 ± 1.6 ms, ventricular TCs) was lowered significantly by TGF-β1 (7.3 ± 0.7 ms, *P* < 0.05, *n* = 6 atrial TCs; 11.1 ± 0.1 ms, *P* < 0.05, *n* = 6 ventricular TCs), but was significantly reversed (11.9 ± 0.3 ms, *P* < 0.05, *n* = 6 atrial TCs; 29.1 ± 0.3 ms, *P* < 0.05, *n* = 6 ventricular TCs) by H_2_S, confirming its modulation of BK_Ca_ channel kinetics in TCs (Fig. 3C).

**Fig 4:**
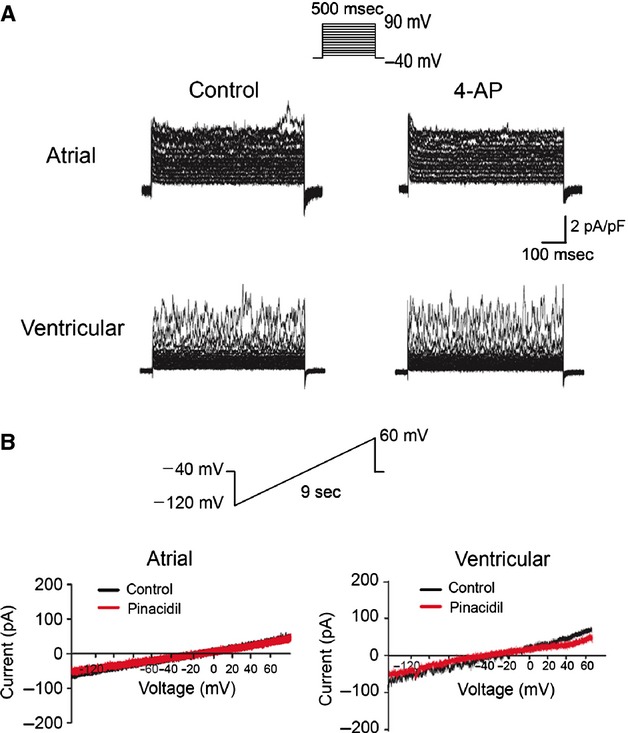
I_to_ and K_ATP_ currents are not detectable in human atrial and ventricular telocytes. (A) Effect of I_to_ inhibitor 4-AP (5 mM) on membrane currents in human atrial and ventricular telocytes showing no noticeable inhibition. (B) Effect of K_ATP_ specific opener Pinacidil on currents elicited by the voltage protocol showing no appreciable enhancement in amplitude.

Similarly, under conditions that elicited total outward K^+^ currents, it was found that the activated currents were insensitive to 4-aminopyridine (4-AP; 0.5 mM) inhibition, indicating the absence of transient outward currents (I_to_) in atrial and ventricular TCs (Fig. 4A). Furthermore, currents elicited from the holding potential of −40 mV that ramped every 9 sec. from −120 mV to +60 mV at 20 mV/sec. and subsequently ramped to −40 mV at −100 mV/sec. were not responsive to 30 μM pinacidil (K_ATP_ specific channel enhancer), suggesting ATP-sensitive K current (K_ATP_) was not present in atrial and ventricular TCs (Fig. 4B). Such differential ion channel profile of TCs further differentiated them from cardiac fibroblasts that showed higher BK_Ca_ current density and presence of I_to_ and K_ATP_ currents that were previously reported by our group 18.

**Fig 5:**
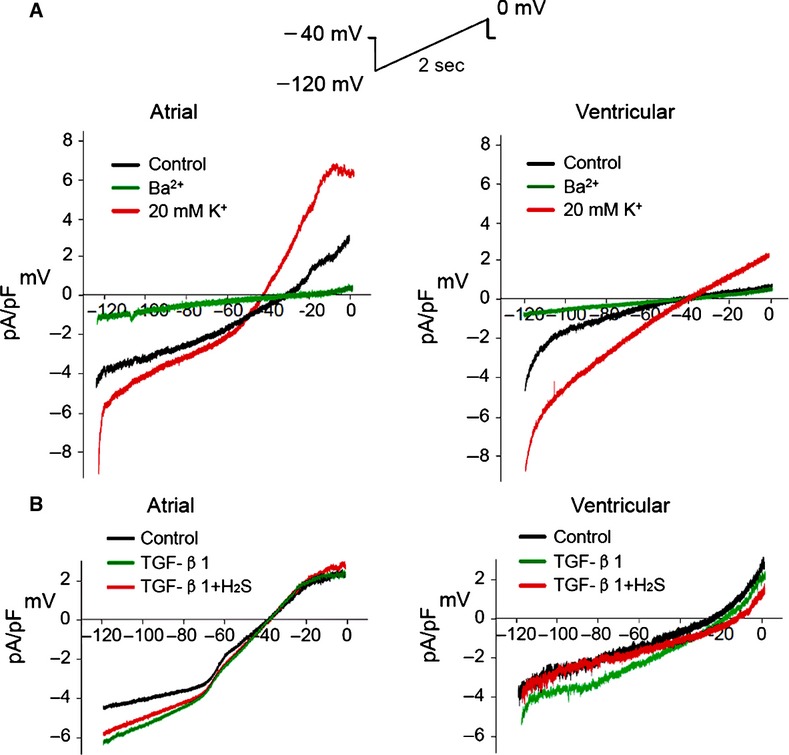
I_Kir_ in human atrial and ventricular telocytes. (A) Effect of Ba^2+^ on membrane current in human atrial and ventricular telocytes. Representative I–V relationships of membrane currents recorded with a 2-sec. ramp protocol (−120 to 0 mV from a holding potential of −40 mV) in 5 mM K^+^ or 20 mM K^+^ and after application of 0.5 mM Ba^2+^. (B) Effect of H_2_S (100 μM) on I_Kir_ currents in the presence of transforming growth factor-β1 (1 ng/ml) in human atrial and ventricular telocytes.

Next, we elicited inwardly rectifying K^+^ currents by depolarizing the TCs with a 2 sec. ramp protocol of −120 to 0 mV from holding potential of −40 mV. Similar to our previous report of IK_ir_ current in human atrial fibroblasts that was attenuated by Ba^2+^ ion 18, addition of Ba^2+^ attenuated the inwardly rectifying currents in both atrial and ventricular TCs, and a 20 mM K^+^ in the bath solution induced a strong increase in the current amplitude, confirming the presence of IK_ir_ inward current in TCs (Fig. 5A). Furthermore, slope conductance 21 of IK_ir_ in atrial and ventricular TCs were 9.5 ± 4.1 pS/pF (*n* = 6) and 3.1 ± 1.2 pS/pF (*n* = 6) in 5.4 mmol/l [K]_o_ respectively. The baseline current density of IK_ir_ at −120 mV (−4.3 ± 0.4 pA/pF, atrial TCs; −4.1 ± 0.3 pA/pF, ventricular TCs) was increased by 1 ng/ml TGF-β1 (−6.4 ± 0.1 pA/pF, *P* < 0.01, *n* = 6 atrial TCs; −5.2 ± 0.1 pA/pF, *P* < 0.01, *n* = 6 ventricular TCs), confirming the functionality of IK_ir_ channel. However, the current density at −120 mV reverted towards baseline (−5.8 ± 0.3 pA/pF, *P* < 0.05, *n* = 6 atrial TCs; −4.3 ± 0.3 pA/pF, *P* < 0.05, *n* = 6 ventricular TCs) in the presence of H_2_S (Fig. 5B).

### H_2_S attenuated TGF-β1-induced KCa1.1 and Kir2.1 expression in cardiac telocytes

**Fig 6:**
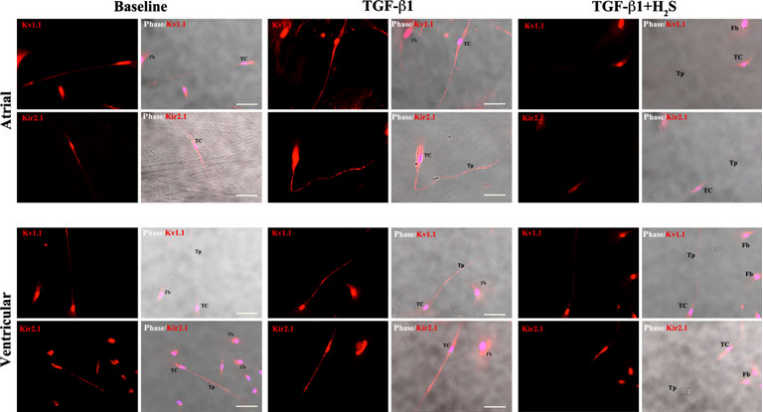
Immunostaining of BK_Ca_ (Kv1.1) and IK_ir_ (Kir2.1) in human atrial (top) and ventricular (bottom) telocytes. Cells were stained with anti-Kv1.1 (red), anti-Kir2.1 (red) and the nuclei were stained using DAPI (blue). H_2_S attenuates the Kv1.1 and Kir2.1 upregulation induced by transforming growth factor-β1. TC: telocyte; Fb: cardiac fibroblast; Tp: telopode. Scale bar corresponds to 50 μm.

Transforming growth factor-β1 is a major mediator of myocardial fibrosis. Our previous study showed that exogenously applied H_2_S significantly attenuated TGF-β1-stimulated KCa1.1/Kv1.1 (responsible for BK_Ca_) and Kir2.1 (responsible for IK_ir_) expression and reduced proliferation of human atrial fibroblasts 18. Consistent with our electrophysiological findings, immunofluorescent staining with anti-Kv1.1 and anti-Kir2.1 antibodies confirmed their presence in cardiac TCs (Fig. 6). Furthermore, stimulation with TGF-β1 enhanced the channel expression in cardiac fibroblasts, but also in the associated TCs. Similarly, pretreatment with H_2_S for 24 hrs reduced the expression levels and organizational distribution of Kv1.1 and Kir2.1 induced by TGF-β1, supporting its inhibitory role on channel expression in cardiac TCs. Contrary to atrial fibroblasts 22 and cardiac fibroblasts in this study, Kv4.3 (responsible for I_to_) expression in TCs was barely above background noise levels (data not shown), which supported the undetectable I_to_ current in our electrophysiological findings.

## Discussion

Telocytes have previously been reported in human epicardium 1. Our results confirmed that ionic active cardiac TCs are present in the human atrial and ventricular myocardium. Concordance with previous report 23, they expressed c-Kit, CD34 and PDGFR-β 20 and exhibited typical TC phenotype such as Tps, podoms and podomers 19. Because of these unique structural characteristics, TCs with moniliform processes were relatively straightforward to distinguish from cultured fibroblasts and other interstitial cells that maintained disparate spindle-like and stellate-shaped cells in culture. On the basis of these criteria, we were able to visually isolate single TCs from human atrial and ventricular fibroblasts and other interstitial cells for their electrophysiological studies. The examined cardiac TCs expressed a subset of ionic currents that was previously reported to be present in human atrial fibroblasts 18. To our knowledge, this is the first study that provides evidence of BK_Ca_ and IK_ir_ channels and functionally active currents in human atrial and ventricular TCs.

To date, only very limited is known about the electrophysiological property of isolated TCs. In human myometrium, patch-clamp recordings of TCs revealed a calcium-dependent hyperpolarization-activated chloride inward current, but absence of L-type calcium channels, which was postulated to modulate myometrial smooth muscle contractions 15. Inwardly rectifying chloride current has been identified in rodent interstitial cells of Cajal (ICC) in regulating gut pacemaker activity 24. Transient outward potassium current that modulated pacemaker potential has also been described in rodent ICC 25. Although transient outward I_to_ and ATP-sensitive K_ATP_ currents were not detected in both the atrial and ventricular TCs in this study, the presence of functionally active BK_Ca_ and hyperpolarized-activated inwardly rectifying IK_ir_ channels and their close proximity to cardiac fibroblasts and cardiomyocytes may suggest a probable role of cardiac TCs within the multicellular framework that facilitates mechano-electrical coupling of the heart.

Myofibroblasts are abundant in fibrosis 26 and have been associated with TGF-β1-mediated atrial fibrillation in rodent 27 and in patients with inflammation-linked atrial remodelling 17. Preventing aberrant proliferation of cardiac fibroblasts and their transformation to myofibroblasts has been an attractive target in limiting fibrosis. Our previous study showed that H_2_S effectively suppressed proliferation of atrial fibroblasts by inhibiting BK_Ca_, IK_ir_ and I_to_ channels 18. Consistently, we found that H_2_S attenuated TGF-β1-induced BK_Ca_ activation and decelerated the transition from closed to open state of the channel, suggesting a role for H_2_S in regulating BK_Ca_ channel kinetic and voltage sensitivity in human cardiac TCs. Furthermore, H_2_S down-regulated the voltage-dependent relationships of IK_ir_ induced by TGF-β1 in the TCs. It is currently unclear about the implications of greater BK_Ca_ (Fig. 3C) and IK_ir_ (Fig. 5B) channel responsiveness observed in the atrial TCs as compared to ventricular TCs when stimulated with TGF-β1. This was despite both TCs exhibited similar current density of BK_Ca_ (Fig. 3B) and IK_ir_ (Fig. 5A) at baseline. Although atrial fibroblasts are known to participate more actively in cardiac fibrosis as compared to ventricular fibroblasts 28,29, it remains to be ascertained if such differential sensitivity of atrial TCs to TGF-β1 stimulation has a role in more aggressive fibrotic response of atrial fibroblasts. Furthermore, the differential disease status of the atrial and ventricular tissue donors (from which TCs were derived in our study), in affecting the observed outcome could not be totally discounted.

In summary, our study shows the presence of functionally competent BK_Ca_ and IK_ir_ channels, but not I_to_ and K_ATP_ channels, in cardiac atrial and ventricular TCs. Their close physical association with cardiac fibroblasts and cardiomyocytes may suggest additional active roles in myocardial physiopathology apart from mere passive structural supporting function in the heart.

## Funding

This study was supported by funding from the National Research Foundation Singapore (NRF-003-CRP-002), the Goh Foundation (Duke-NUS-GCR/2013/008) and Biomedical Research Council Singapore (BMRC 13/1/96/19/86) to W.S.

## Conflict of interest

The authors confirm that there are no conflicts of interest.

## Supporting information

Figure S1 Double immunofluorescent staining of human atrial and ventricular telocytes demonstrating positivity for CD34 and c-Kit. Scale bar corresponds to 10 μm.
