# Supplementary figures and images for: Electrophysiology of human cardiac atrial and ventricular telocytes
Source: J Cell Mol Med. 2014 Jan 28;18(2):355–62. doi: 10.1111/jcmm.12240 (PMC3930421; doi:10.1111/jcmm.12240)

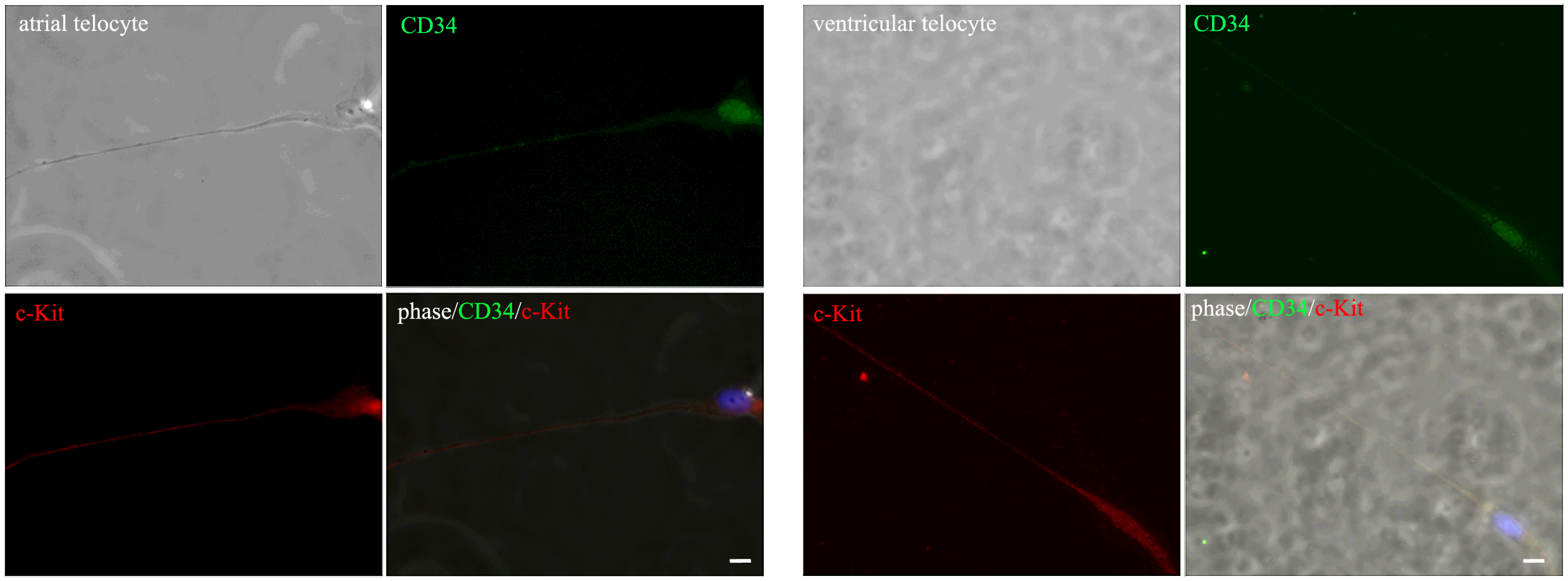

Supplement: Figure S1 — Double immunofluorescent staining of human atrial and ventricular telocytes demonstrating positivity for CD34 and c-Kit. Scale bar corresponds to 10 μm. [file jcmm0018-0355-sd1.tif]
